# Phenolic Profiling of *Duchesnea indica* Combining Macroporous Resin Chromatography (MRC) with HPLC-ESI-MS/MS and ESI-IT-MS

**DOI:** 10.3390/molecules201219859

**Published:** 2015-12-15

**Authors:** Mingzhi Zhu, Xia Dong, Mingquan Guo

**Affiliations:** 1 Key Laboratory of Plant Germplasm Enhancement and Specialty Agriculture, Wuhan Botanical Garden, Chinese Academy of Sciences, Wuhan 430074, China; mzzhucn@hotmail.com (M.Z.); dongfuxia623@163.com (X.D.); 2 State Key Laboratory of Quality Research in Chinese Medicine, Macau Institute for Applied Research in Medicine and Health, Macau University of Science and Technology, Macau, China; 3 Sino-Africa Joint Research Center, Chinese Academy of Sciences, Wuhan 430074, China

**Keywords:** *Duchesnea indica*, phenolic profiling, HPLC, mass spectrometry

## Abstract

*Duchesnea indica* (*D. indica*) is an important traditional Chinese medicine, and has long been clinically used to treat cancer in Asian countries. It has been described previously as a rich source of phenolic compounds with a broad array of diversified structures, which are the major active ingredients. However, an accurate and complete phenolic profiling has not been determined yet. In the present work, the total phenolic compounds in crude extracts from *D. indica* were enriched and fractionated over a macroporous resin column, then identified by HPLC-ESI-MS/MS and ESI-IT-MS (ion trap MS). A total of 27 phenolic compounds were identified in *D*. *indica*, of which 21 compounds were identified for the first time. These 27 phenolic compounds encompassing four phenolic groups, including ellagitannins, ellagic acid and ellagic acid glycosides, hydroxybenzoic acid and hydroxycinnamic acid derivatives, and flavonols, were then successfully quantified using peak areas against those of the corresponding standards with good linearity (R^2^ > 0.998) in the range of the tested concentrations. As a result, the contents of individual phenolic compounds varied from 6.69 mg per 100 g dry weight (DW) for ellagic acid to 71.36 mg per 100 g DW for brevifolin carboxylate. Not only did this study provide the first phenolic profiling of *D. indica*, but both the qualitative identification and the subsequent quantitative analysis of 27 phenolic compounds from *D. indica* should provide a good basis for future exploration of this valuable medicinal plant.

## 1. Introduction

*Duchesnea indica*, distributed widely throughout Asia, Europe and America, is a perennial plant that belongs to the Rosaceae family [1,2,3]. It has been used as a traditional herbal medicine in Asia for thousands of years, mainly for the treatment of leprosy, congenital fever, tissue inflammation, haematemesis, and cancer, among other uses [3]. Nowadays, it is clinically used for cancer therapy alone or as a main ingredient in Chinese herbal medicine formulas for the treatment of cancers, especially gynecological cancers [3]. Several phenolic compounds, including phenolic acids, ellagic acids and flavonoids [4,5,6], have been isolated from genus *Duchesnea*, and further pharmacological studies have shown that phenolic compounds are the major active ingredients [3,4,7]. It was reported that some phenolic compounds isolated from *D. chrysantha*, especially brevifolin carboxylic acid, showed a strong cytotoxic activity to PC_14_ human lung adenocarcinoma cells and MKN_45_ human gastric adenocarcinoma cells [4]. The study of the anticancer mechanisms showed that the phenolic extracts of *D. indica* significantly inhibited SKOV-3 ovarian cancer cell proliferation through induction of apoptosis via mitochondrial pathway and arresting cell cycle progression in the S phase [3]. In another study, the phenolic extracts of *D. indica* inhibited *in vitro* and *in vivo* growth of cervical cancer through induction of apoptosis and cell-cycle arrest [7]. In addition, a variety of other biological activities, including anti-oxidative [8,9], immunomodulatory [10], and anti-inflammatory activities [11,12], have been documented in recent years.

Phenolic compounds, which are nearly ubiquitous in plants, encompass a broad spectrum of molecules that contain an aromatic group and one or more hydroxyl groups on the aromatic ring [13], ranging from simple phenolic acids (such as derivatives of benzoic acid or cinnamic acid) to the complex flavonoids and tannins families [14]. In the past few years, great focus has been put on the phenolic compounds due to their various biological activities, such as reduction in the incidence of some degenerative diseases like cancer and diabetes [15,16], reduction in risk factors of cardiovascular diseases [17], and anti-oxidant [18], anti-allergenic [19], anti-inflammatory [20], and antimicrobial activities [21]. Although some phenolic compounds in *D. indica* have been isolated and identified by nuclear magnetic resonance spectroscopy (NMR) [4,5,6], extended profiling of phenolic compounds from *D. indica* remains a big challenge [22]. In addition, it is well accepted that herbal medicines exert their curative effects through multiple components acting on multiple-target sites [23]. Thus, development of reliable and highly sensitive methods to identify multiple bioactive components for their comprehensive quality control has become a rational strategy [24].

In this context, we strived to develop a highly effective method for the simultaneous separation and comprehensive analysis of phenolic compounds from the extracts of *D. indica*. To achieve this, at least two tough challenges must be addressed. Firstly, due to the fact phenolic compounds usually exist in complex plant extracts together with hundreds of other phytochemicals in very minute amounts, the direct analysis of phenolic compounds from complex matrices is often a very tough task. Thus, an efficient method for pre-concentration and purification of total phenolic compounds from complex plant extracts is a prerequisite for this type of work. Fortunately, the macroporous resin chromatography (MRC), with its properties of high adsorption capacity, good stability and easy regeneration, is one of the most efficient methods to enrich some specific types of bioactive components from crude raw herbal material extracts, so it was thus successfully developed to enrich the total phenolic compounds in crude extracts from *D. indica*. Secondly, due to the structural diversity of phenolic compounds, it is also very difficult to conduct a comprehensive detection of phenolic compounds from the plant since different types of structures often exhibit different detection sensitivities to common detectors, such as the UV detector used in most LC equipment. To overcome this obstacle, two characteristic wavelengths were selected for the detection based on the structural properties of the phenolic compounds of interest: 260 nm for ellagitannins, ellagic acid, ellagic acid glycosides, and 360 nm for hydroxybenzoic acid derivatives, hydroxycinnamic acid derivatives, as well as flavonols [25]. As a result, the use of two characteristic UV wavelengths for the detection of phenolic compounds significantly expanded the detection range. To this end, our hypothesis was that combination of MRC with HPLC-UV and LC-MS could be successfully developed for simultaneous enrichment and analysis of phenolic compounds from *D. indica*, and some compounds which are difficult to be identified by MS/MS could be further identified by ion trap MS. Once all of the phenolic components from *D. indica* were successfully identified, the subsequent quantitative analysis of these components could be conducted based on a comparison of their peak areas against the corresponding phenolic compound standards. To the best of our knowledge, this is the first comprehensive phenolic profiling of *D. indica* providing the most detailed information on its phenolic compounds, and offers valuable information for quality control purposes or further pharmaceutical studies on this valuable plant.

## 2. Results and Discussion

### 2.1. Identification of Phenolic Compounds in D. indica

**Figure 1:**
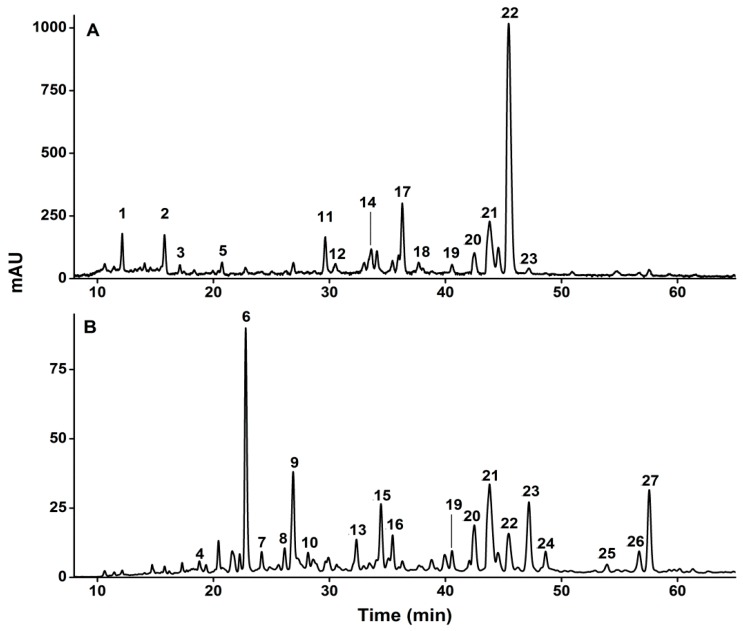
The HPLC profiles of phenolic compounds in fraction I from *D. indica* recorded at 260 nm (**A**) and 360 nm (**B**). The peak numbers correspond to those used in Table 1.

Phenolic compounds in *D. indica* were identified based on LC-MS/MS data and/or further confirmed by comparing their LC-MS/MS spectra with the corresponding standards. In order to conduct a comprehensive analysis of phenolic compounds from this plant, two characteristic wavelengths were selected for the detection based on the structural properties of the compounds of interest: 260 nm for ellagitannins, ellagic acid, and ellagic acid glycosides, and 360 nm for hydroxybenzoic acid derivatives, hydroxycinnamic acid derivatives, as well as flavonols [25], and the HPLC profiles of the phenolic compounds in fraction I at 260 and 360 nm are shown in Figure 1A,B, respectively.

The total phenolic components were found to be enriched in fraction I. The chromatographic profiles of fractions II and III are not shown because no peaks were detected in those two fractions under the same experimental conditions, which further implied that the phenolic components had been successfully enriched and eluted in fraction I. In more details, the 27 phenolic compounds corresponding to the numbers in Figure 1 are summarized in Table 1. The details on the identification and verification of these compounds are described below.

**Table 1:** Qualitative and quantitative analysis of 27 phenolic compounds in fraction I from *D. indica.*

Peak	t_R_ (min) ^a^	[M − H]^−^ (*m*/*z*)	MS/MS Ions (*m*/*z*)	MS^3^ Ions (*m*/*z*)	Compounds	Contents (mg/100 g DW)
1	12.1	783	481, 301 ^b^	257, 229, 185	bis-HHDP-glucose ^c^	29.10
2	15.8	783	481, 301 ^b^	257, 229, 185	bis-HHDP-glucose ^c^	33.85
3	17.1	783	481, 301 ^b^	257, 229, 185	bis-HHDP-glucose ^c^	15.90
4	18.8	337	191, 173, 163, 155		*p*-coumaroyl quinic acid ^c^	15.97
5	20.8	633	481, 301 ^b^	257, 229, 185	galloyl-HHDP-glucose ^c^	25.33
6	22.8	291	247, 219, 191		brevifolin carboxylate ^d^	71.36
7	24.2	179	135, 134		caffeic acid ^d^	14.74
8	26.2	297	179, 161, 135, 117		caffeic acid derivate ^c^	17.64
9	26.9	637	461, 285 ^b^	93	kaempferol *O*-diglucuronide ^c^	15.04
10	28.2	337	191, 173, 163, 155		*p*-coumaroyl quinic acid ^c^	14.74
11	29.7	783	633, 481, 301 ^b^	257, 229, 185	bis-HHDP-glucose ^c^	13.20
12	30.5	783	633, 481, 301 ^b^	257, 229, 185	bis-HHDP-glucose ^c^	11.93
13	32.3	247	219, 191		Brevifolin ^c^	21.71
14	33.6	935	633, 301 ^b^	257, 229, 185	galloyl-bis-HHDP-glucose ^c^	16.17
15	34.4	305	273, 245, 229		methyl brevifolin carboxylate	43.56
16	35.5	563	503, 473, 383, 353		apigenin 6-*C*-arabinosyl-8-*C*-glucoside (isoschaftoside) ^d^	10.13
17	36.3	783	633, 481, 301 ^b^	257, 229, 185	bis-HHDP-glucose ^c^	26.24
18	37.7	783	633, 481, 301 ^b^	257, 229, 185	bis-HHDP-glucose ^c^	14.87
19	40.6	433	301 ^b^	257, 229, 185	ellagic acid pentoside ^c^	11.18
20	42.5	433	301 ^b^	257, 229, 185	ellagic acid pentoside ^c^	12.64
21	43.8	301	257, 229, 185		ellagic acid ^d^	6.69
22	45.4	935	633, 301 ^b^	257, 229, 185	galloyl-bis-HHDP-glucose ^c^	48.89
23	47.2	935	633, 301 ^b^	257, 229, 185	galloyl-bis-HHDP-glucose ^c^	17.50
24	48.6	477	301 ^b^, 179, 151	179 151	quercetin *O*-glucuronide ^c^	10.06
25	53.9	593	285 ^b^	93	kaempferol *O*-robinobioside ^c^	12.15
26	56.7	447	284, 255 ^b^, 227	93	kaempferol 3-*O*-glucoside (astragalin) ^d^	17.55
27	57.6	461	285 ^b^	93	kaempferol *O*-glucuronide ^c^	25.45
Total ellagitannins						252.98
Total ellagic acid and ellagic acid glycosides						30.52
Total hydroxybenzoic acid and hydroxycinnamic acid derivatives						199.73
Total flavonols						90.39

^a^ Peak numbers and retention times refer to Figure 1; ^b^ These ions were further fragmented to yield the MS^3^ data; ^c^ Identified based on the published literature; ^d^ Identified with the corresponding standards.

#### 2.1.1. Ellagitannins

**Figure 2:**
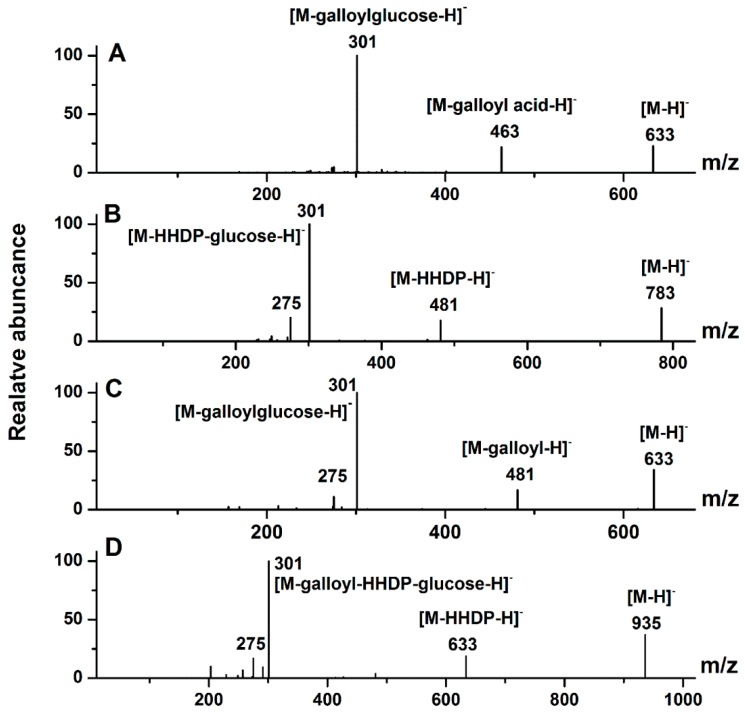
The MS/MS spectra of the standard of corilagin and some representative ellagitannins in *D. indica*: (**A**) corilagin; (**B**) peak 1; (**C**) peak 5; (**D**) peak 14. The peak numbers correspond to those used in Table 1.

The major phenolic compounds found in *D. indica* belonged to the ellagitannins family (Table 1). Ellagitannins are hydrolyzable tannins, esterified with hexahydroxydiphenic acid (HHDP) and a polyol, usually glucose [26,27]. Typical neutral losses of ellagitannins during MS fragmentation are galloyl (152 Da), gallic acid (170 Da), HHDP (302 Da), galloylglucose (332 Da), HHDP glucose (482 Da), and galloyl-HHDP-glucose (634 Da) residues [28]. A key reaction of ellagitannins is the release of the bislactone and formation of a HHDP ester group, which undergo rapid, facile and unavoidable lactonization to yield ellagic acid [29]. The multistage tandem MS using ion trap mass spectrometer was used to distinguish between conjugates of quercetin and ellagic acid since they produce identical deprotonated ions (*m*/*z* 301) in the MS/MS spectra. However, in the MS^3^ spectrum, the deprotonated quercetin ion further forms the characteristic ions at *m*/*z* 179 and 151, whereas the equivalent deprotonated ellagic acid ion yields ions at *m*/*z* 257, 229 and 185 [30]. As examples, the MS/MS spectra of some representative ellagitannins are shown in Figure 2. Furthermore, we first used a standard of corilagin to validate the fragmentation features of ellagitannins described above (Figure 2A). The compound corilagin showed a [M − H]^−^ ion at *m*/*z* 633, and MS/MS fragment ions at *m*/*z* 463 [M − 170 − H]^−^ (loss of galloyl acid), and 301 [M − 332 − H]^−^ (loss of galloylglucose). The neutral losses of galloyl acid, galloylglucose and the characteristic ion at *m*/*z* 301 for corilagin coincide with the fragmentation pathway of ellagitannins. In this way, we can tentatively identify the structures of ellagitannins in *D. indica* despite the lack of some commercially available standards. As a result, peaks 1, 2, 3, 5, 11, 12, 14, 17, 18, 22 and 23 were tentatively identified as ellagitannins due to their similar fragmentation features based on their LC-MS/MS data as shown in Table 1, especially their key MS/MS fragment at *m*/*z* 301, and further MS^3^ fragments (*m*/*z* 257, 229 and 185) showing that the ion at *m*/*z* 301 was deprotonated ellagic acid. In addition, some high molecular weight ellagitannins usually exhibited doubly charged quasi-molecular ions in the ESI-MS data [30]. However, in the present work all peaks were determined to be singly charged by “Zoom scan” analysis [31]. As shown in Table 1 and Figure 2B, peaks 1, 2, 3, 11, 12, 17 and 18 were isomeric compounds, with the [M − H]^−^ ion at *m*/*z* 783, yielding main fragment ions at *m*/*z* 481 [M − 302 − H]^−^ (loss of HHDP) and 301 [M − 482 − H]^−^ (loss of HHDP-glucose), whose fragmentation pattern corresponds to a bis-HHDP-glucose structure [32,33]. Peaks 1, 2, 3, 11, 12, 17 and 18 were thus identified as bis-HHDP-glucoses, presumably casuariin or pedunculagin isomers, which were previously reported in walnut [33]. However, these seven ellagitannins were identified from *D. indica* in our study for the first time.

Peak 5 and the corilagin standard showed the same ions at *m*/*z* 633 [M − H]^−^ and 301 [M − 332 − H]^−^ (loss of galloylglucose), while peak 5 yielded a minor signal at *m*/*z* 481 [M − 152 − H]^−^ (loss of a galloyl residue), and corilagin yielded a minor signal at *m*/*z* 463 [M − 170 − H]^−^ (loss of galloyl acid) (Figure 2A,C). Loss of a galloyl residue for peak 5 suggested that the galloyl unit was most probably bonded via a *meta*-depside bond, and not attached directly to the glucose core because a galloyl bond via a *meta*-depside bond is more cleavable [28,34]. On the contrary, the galloyl unit of corilagin was attached directly to the glucose. Thus, the compound corresponding to peak 5 was identified as galloyl-HHDP-glucose, which was previously reported in strawberry [25,28], but was here identified in *D. indica* for the first time.

Peaks 14, 22 and 23 were also isomeric compounds with the same [M − H]^−^ ion at *m*/*z* 935 and MS/MS fragment ions at *m*/*z* 633 [M − 302 − H]^−^ (loss of HHDP) and 301 [M − 634 − H]^−^ (loss of galloyl-HHDP-glucose) (Figure 2D) [25]. Based on their LC-MS/MS data, they can be tentatively identified as galloyl-bis-HHDP-glucoses. Galloyl-bis-HHDP-glucose, probably casuarinin or casuariin, is a basic unit of many ellagitannins, such as sanguiin H-6 and lambertianin C [35]. These three isomeric ellagitannins were also found from *D. indica* for the first time. Altogether, eleven ellagitannins were identified from *D. indica* for the first time, including peaks 1, 2, 3, 5, 11, 12, 14, 17, 18, 22 and 23 as shown in Figure 1.

#### 2.1.2. Ellagic Acid and Ellagic Acid Glycosides

**Figure 3:**
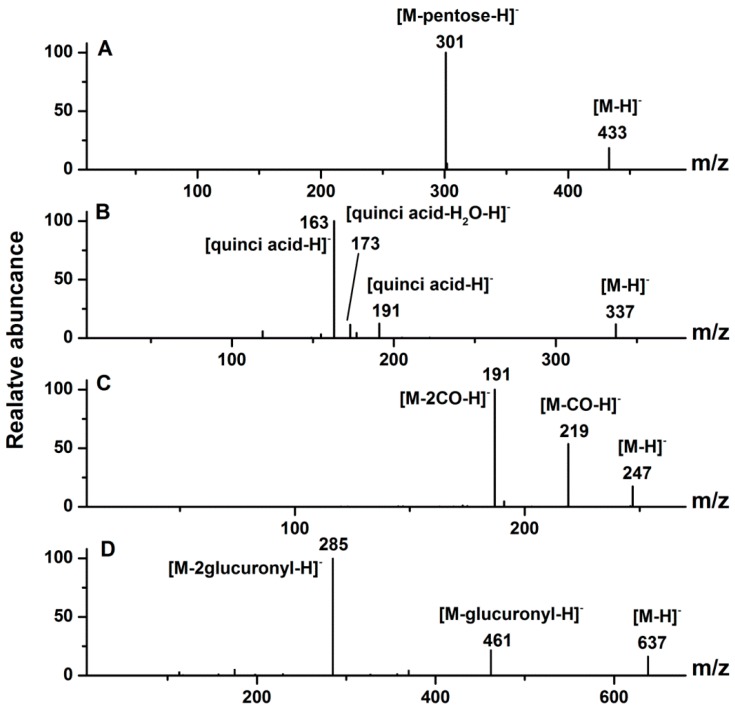
The MS/MS spectra of representative ellagic acid glycoside ((**A**) peak 19), hydroxybenzoic acid derivative ((**B**) peak 4), hydroxycinnamic acid derivative ((**C**) peak 13), and flavonol ((**D**) peak 9). The peak numbers correspond to those used in Table 1.

In the case of ellagic acid and ellagic acid glycosides, a representative MS/MS spectrum of peak 19 is shown in Figure 3A. Actually, peaks 19 and 20 had the same [M − H]^−^ ion at *m*/*z* 433 and MS/MS fragment ion at *m*/*z* 301 [M − 132 − H]^−^ (loss of a pentose), corresponding to the deprotonated ellagic acid or quercetin. MS^3^ fragment ions for both ions at *m*/*z* 301 were at *m*/*z* 257, 229 and 185, indicated peaks 19 and 20 were sugar conjugates of ellagic acid. Peaks 19 and 20 were thus tentatively identified as ellagic acid pentosides, which were previously reported in Muscadine grapes [36], but were identified from *D. indica* for the first time in this work.

As for another peak 21, the [M − H]^−^ ion at *m*/*z* 301 was observed, and its corresponding MS/MS fragment ions at *m*/*z* 257, 229, and 185, which are typical fragment ions of ellagic acid [28]. By comparing its LC-MS/MS spectra with the corresponding ellagic acid standard, this peak was confirmed as ellagic acid, which was previously found in *D. chrysantha* [6].

#### 2.1.3. Hydroxybenzoic Acid and Hydroxycinnamic Acid Derivatives

As for hydroxybenzoic acid derivatives from *D. indica*, a representative MS/MS spectrum of peak 4 is shown in Figure 3B. Peaks 4 and 10 showed the same [M − H]^−^ ion at *m*/*z* 337 and fragment ions at *m*/*z* 191 [quinic acid-H]^−^ (losing a coumarin residue of 146 Da) and 163 [*p*-coumaric acid-H]^−^ (losing a quinic acid residue of 174 Da), which coincided with the fragmentation pattern of quinic acid derivatives containing one *p*-coumaric acid moiety [37]. On the basis of the mass spectra and previously published data, peaks 4 and 10 were tentatively identified as *p*-coumaroyl quinic acids [37,38], which were identified from *D. indica* for the first time in this work.

Peak 7 had a [M − H]^−^ ion at *m*/*z* 179, and MS/MS fragmentation ion at *m*/*z* 135 due to the loss of carbon dioxide, which coincided with the fragmentation pattern of caffeic acid [39]. Peak 7 was thus identified as caffeic acid, confirmed by comparison with the corresponding standard. Caffeic acid was previously separated from *D. chrysantha* [4]. Peak 8 had a [M − H]^−^ ion at *m*/*z* 297, and MS/MS fragmentation ions at *m*/*z* 179 [caffeic acid-H]^−^ (loss of a 118 Da moiety) and 135 [caffeic acid-CO_2_-H]^−^ indicating the presence of a caffeic acid moiety [39]. Thus, peak 8 was tentatively identified as a caffeic acid derivative [40], which was also identified from *D. indica* for the first time in this work.

Peak 6 exhibited the [M − H]^−^ ion at *m*/*z* 291 and fragment ions at *m*/*z* 247, 219 and 191, which coincided with the MS/MS data of brevifolin carboxylate [41]. By comparing its LC-MS/MS data with the corresponding standard, peak 6 was confirmed as brevifolin carboxylate [42]. As shown in Figure 3C, peak 13 showed the [M − H]^−^ ion at *m*/*z* 247 and MS/MS fragmentation ions at *m*/*z* 219, 191. The MS/MS spectrum of peak 13 was similar to that of peak 6, suggesting that the compound corresponding to peak 13 may result from the cleavage of the ester bond and subsequent loss of the CO_2_ group from brevifolin carboxylate [41]. Peak 15 showed the [M − H]^−^ ion at *m*/*z* 305 and fragment ions at *m*/*z* 273, 245 and 229, which suggested a methyl ester derivative of brevifolin carboxylate. Peaks 13 and 15 were thus identified as brevifolin and methyl brevifolin carboxylate, respectively. Brevifolin carboxylate, brevifolin and methyl brevifolin carboxylate were previously reported in *D. indica* [42].

#### 2.1.4. Flavonols

In terms of flavonols from *D. indica*, a representative MS/MS spectrum of peak 9 is shown in Figure 3D. With a quasi-molecular ion at *m*/*z* 637 [M − H]^−^, and fragment ions at *m*/*z* 461 [M − H − 176]^−^ (loss of one glucuronyl unit) and 285 [M − H − 176 − 176]^−^ (loss of two glucuronyl units), peak 9 could be thus deduced as a diglucuronic acid glycoside and the aglycone was kaempferol. As a result, peak 9 was thus tentatively identified as kaempferol *O-*diglucuronide, which was previously found in lotus leaf [43]. Similarly, peak 27 was identified as kaempferol *O-*glucuronide by the quasi-molecular ion at *m*/*z* 461 [M − H]^−^ and the MS/MS fragment at *m*/*z* 285 [M − H − 176]^−^ (loss of one glucuronyl unit) [44]. Both flavonols were identified from *D. indica* for the first time in this study.

Peak 16 showed fragment ions at *m*/*z* 563 [M − H]^−^, 503 [M − H − 60]^−^, 473 [M − H − 90]^−^, 383 [M − H − 180]^−^ (A [aglycone: apigenin (Mw 270)] + 113), and 353 [M − H − 210]^−^ (A + 83). The neutral losses of 60 Da, 90 Da and 180 Da coincided with that reported by Ferreres *et al.* [45] for di-*C-*hexosyl-pentosyl flavone and suggested the presence of apigenin (270 Da) + hexose (162 Da) + pentose (132 Da). Hence, peak 16 was identified as apigenin 6*-C-*arabinosyl-8*-C*-glucoside (isoschaftoside) by comparing its LC-MS/MS data with the standard. It was also identified from *D. indica* for the first time in this work.

Peak 24 exhibited a precursor ion at *m*/*z* 477 [M − H]^−^ with a MS/MS fragment ion at *m*/*z* 301 [M − H − 176]^−^, and further MS^3^ fragmentation of the ion at *m*/*z* 301 matched that of quercetin standard (*m*/*z* 179 and 151). Peak 24 was thus identified as quercetin *O-*glucuronide, previously reported in red wines [46], but found in *D. indica* for the first time in this study.

Peak 25 produced a quasi-molecular ion at *m*/*z* 593 [M − H]^−^, and an aglycone ion at *m*/*z* 285 [M − H − 308]^−^, corresponding to a neutral loss of two sugar moieties (hexose (162 Da) + deoxyhexose (146 Da)). Peak 25 was made up of flavonol diglycosides and one aglycone (kaempferol). For flavonol *O-*diglycosides, the mass spectrometric behaviors of diglycosides are notably different depending on the linkage between the two monosaccharides. Radical aglycone ions ([Y_0_ − H]^−^) tend to be generated in the case of a C1 → C2 linkage between the two monosaccharides, while the predominant deprotonated aglycone ion (Y_0_^−^) is indicative of a C1 → C6 linkage [47]. The high abundance of Y_0_^−^ ion at *m*/*z* 285 for peak 25 indicated that the interglycosidic linkage between the two monosaccharides in this compound was C1 → C6. Peak 25 was thus tentatively identified as kaempferol *O-*robinobioside [48], previously reported in *D. indica* [42].

Peak 26 showed a [M − H]^−^ ion at *m*/*z* 447, and a predominant fragment ion at *m*/*z* 284 [Y_0_ − H]^−^. For flavonol mono-*O*-glycosides, glycosylation takes place at the 3-position if the relative abundance of the [Y_0_ − H]^−^ ion is significantly higher than that of the Y_0_^−^ ion, and the situation is reversed when glycosylation happens at the 7-position [47]. The high abundance of the [Y_0_ − H]^−^ ion for peak 26 indicated that glycosylation happened at the 3-position. Peak 26 was thus identified as kaempferol 3-*O*-glucoside (astragalin) and this was confirmed by comparison with the corresponding standard. This compound was found in *D. indica* for the first time in this study.

Overall, 27 phenolic compounds were identified in *D. indica* in this study, of which 21 phenolic compounds, excluding brevifolin carboxylate (peak 6), caffeic acid (peak 7), brevifolin (peak 13), methyl brevifolin carboxylate (peak 15), ellagic acid (peak 21), and kaempferol *O*-robinobioside (peak 25), were identified for the first time.

### 2.2. Quantification of Phenolic Compounds in D. indica

The individual phenolic compounds in *D. indica* could be quantified based on their LC peak areas against the corresponding standard phenolic compounds. As shown in Table 2, the calibration curves of the five representative phenolic standards showed good linearity (R^2^ ≥ 0.9982), which implied that the quantification of individual phenolic compounds from *D. indica* could be achieved. Meanwhile, the corresponding limits of detection (LOD) and quantification (LOQ) were all determined at a signal-to-noise ratio (S/N) of about 3 and 10, respectively. As a result, the lowest LOD and LOQ were obtained for kaempferol 3-*O*-glucoside (0.06 and 0.21 μg/mL). Since all of the 27 phenolic compounds can be structurally classified into five groups, the five representative standards are used for the quantification of individual phenolic compounds in the same group from *D. indica*. In this work, compound **16** was quantified using the standard of apigenin 6-*C*-arabinosyl-8-*C*-glucoside. The ellagitannins, ellagic acid and ellagic acid glycosides, hydroxybenzoic acid and hydroxycinnamic acid derivatives, and other flavonols were quantified using corilagin, ellagic acid, caffeic acid, and kaempferol 3-*O*-glucoside, respectively.

**Table 2:** Liner equation, correlation coefficients, limits of detection, and limits of quantification of five representative phenolic standards.

Compounds	Liner Equation (μg/mL)	R^2^	Linear Range (μg/mL)	LOD ^a^ (μg/mL)	LOQ ^b^ (μg/mL)
Caffeic acid	y = 8.56x − 34.67	0.9997	0.94–588	0.17	0.57
Apigenin 6-*C*-arabinosyl-8-*C*-glucoside	y = 16.38x − 37.08	0.9998	0.69–430	0.07	0.24
Ellagic acid	y = 14.53x − 25.08	0.9982	0.90–560	2.24	7.47
Corilagin	y = 17.91x − 59.47	0.9997	0.82–510	0.19	0.65
Kaempferol 3-*O*-glucoside	y = 21.39x − 59.04	0.9998	0.80–500	0.06	0.21

^a^ LOD = limit of detection, S/N = 3; ^b^ LOQ = limit of quantitation, S/N = 10.

As shown in Table 1, *D. indica* was rich in ellagitannins, and the total ellagitannins content was 252.98 mg/100 g DW (dry weight), followed by the total hydroxybenzoic acid and hydroxycinnamic acid derivatives content at about 199.73 mg/100 g DW, in which brevifolin carboxylate was the dominant one. The total flavonols content was 90.39 mg/100 g DW, and the total ellagic acid and ellagic acid glycosides content was the lowest at 30.52 mg/100 g DW. These quantitative data on individual phenolic components are very useful and may play an important role in the quality control process or future exploration of *D. indica* as a valuable medicinal plant.

## 3. Materials and Methods

### 3.1. Chemicals and Materials

The standard of kaempferol 3-*O*-glucoside was purchased from Shanghai Tauto Biotech (Shanghai, China). Five compounds (brevifolin carboxylate, caffeic acid, apigenin 6-*C*-arabinosyl-8-*C*-glucoside, corilagin and ellagic acid) were isolated from *D. indica* in our laboratory, and used as standards after elucidating their chemical structures by NMR. HPLC-grade solvents (acetonitrile and formic acid) were purchased from Sigma-Aldrich Corp. (Shanghai, China). HPLC-grade water was obtained using a Milli-Q System (Millipore, Billerica, MA, USA). Other chemicals of analytical grade were obtained from China Medicine (Group) Shanghai Chemical Reagent Corp. (Shanghai, China). Millipore membranes (0.22 um) were purchased from Jinteng Experiment Equipment Corp. (Tianjin, China). AB-8 macroporous resin (pore size 130–140 Å) was purchased from an industrial chemical company affiliated to Nan Kai University (Tianjin, China). Whole plants of *D. indica* were collected from Wuhan Botanical Garden of Chinese Academy of Sciences (WBGCAS, Wuhan, China) in October 2013 and identified by Professor Mingxi Jiang from WBGCAS. A voucher specimen (Voucher No. DC201301) has been deposited at The Key Laboratory of Plant Germplasm Enhancement and Specialty Agriculture, WBGCAS. The whole plant of *D. indica* was dried at 45 °C, and then stored at 4 °C until use.

### 3.2. Extraction and Fractionation of Extracts from D. indica

Whole plants of *D. indica* were powdered and a sample (100 g) was extracted in an ultrasonic bath (SB25-12DTDN, 500 × 300 × 250 mm, SCIENTZ, Ningbo, China) at room temperature for 40 min with 1000 mL of 70% ethanol. After repeating the extraction three times, the extracts were combined and filtered, and the supernatants were evaporated under reduced pressure and lyophilized to afford dark-green residues. These residues were dissolved in H_2_O (50 mL) and subjected to liquid/liquid partitioning with petroleum ether (b.p. 60–90 °C) in order to remove chlorophyll. Later, the lower layer was evaporated, and fractionated by AB-8 macroporous resin chromatography (45 cm × 5.3 cm). The column was washed with distilled water to remove water soluble impurities (sugar, protein, and other water-soluble molecules), and then eluted successively with 30%, 50%, 70%, and 90% ethanol. The eluates were named fractions I, II, III and IV, respectively. All the fractions were concentrated to yield an aqueous residue, which was freeze-dried, then redissolved in 50% methanol and stored at 4 °C until use.

### 3.3. HPLC-UV Analysis of Phenolic Compounds

HPLC-UV analysis of phenolic compounds was carried out using a Thermo Accela 1250 U-HPLC system (Thermo Fisher Scientific, San Jose, CA, USA) equipped with a binary solvent pump, column oven, auto-sampler and UV detector. A 10 μL aliquot of each sample solution was injected and analyzed on a PACK ODS-A column (250 mm × 4.6 mm, 5 μm, YMC, Kyoto, Japan). The separation was conducted at 30 °C (column temperature) using a gradient elution method with 0.5% formic acid in distilled water (solvent A) and acetonitrile (solvent B). The solvent gradient in volumetric ratios was set as follows: 0–5 min at 95% A; 5–10 min from 95% A to 90% A; 10–68 min from 90% A to 78% A. The flow rate was 0.8 mL/min and the effluents were monitored at 260 nm and 360 nm at the same time.

### 3.4. Identification of Phenolic Compounds

#### 3.4.1. ESI-MS/MS analysis

Phenolic compounds were identified using a Thermo Accela 1250 U-HPLC system with a UV detector coupled to a triple quadropole mass spectrometer (TSQ Quantum Access MAX, Thermo Fisher Scientific). Electrospray ionization (ESI) MS analysis was applied in the negative ion (NI) mode. The operation conditions were set as follows: capillary temperature, 350 °C; vaporizer temperature, 300 °C; sheath gas (N_2_) pressure, 40 arbitrary units; auxiliary gas (N_2_) pressure, 10 arbitrary units; spray voltage, 3 kV. The mass spectra were recorded in the mass range from *m*/*z* 150 to 2000. The MS/MS spectra were obtained using the Data-Dependent scan mode and the collision energy was set as following: collision energy (CE), 10 V; collision energy grad (CE grad), 0.035 V/m.

#### 3.4.2. ESI-IT-MS analysis

To gather multistage tandem MS information about several chromatographic peaks, the fractions corresponding to these chromatographic peaks were manually collected following the conditions discussed above. The collected HPLC fractions were dissolved in methanol and directly injected into an ion trap mass spectrometer (IT-MS, LCQ, Finnigan, San Jose, CA, USA) via a syringe pump at a flow rate of 3 μL/min. Optimal ESI conditions in the NI mode were set as follows: sheath gas (N_2_) pressure, 10 arbitrary units; spray voltage, 4.5 kV; capillary temperature, 275 °C; capillary voltage, −10 V and tube lens voltage, −50 V. MS/MS and MS^n^ experiments were performed on selected precursor ions with optimized collision energy.

### 3.5. Quantification of Phenolic Compounds

The five representative standards (kaempferol 3-*O*-glucoside, caffeic acid, apigenin 6-*C*-arabinosyl-8-*C*-glucoside, corilagin and ellagic acid) were accurately weighed, dissolved in 50% methanol, and then diluted to obtain a series of standard solutions with different concentrations. Subsequently, the calibration curves of these five compounds can be established using LC at 260 nm or 360 nm. At least six concentrations of the five standard solutions were analyzed in triplicate. The limits of detection (LOD) and quantification (LOQ) under the present chromatographic conditions were determined at a signal-to-noise ratio (S/N) of about 3 and 10, respectively. Finally, quantitative analysis of individual phenolic compounds could be conducted based on their LC peak areas against those of the corresponding standard phenolic compounds. In this study, individual phenolic compounds are presented as mg per 100 g DW.

## 4. Conclusions

To initiate a comprehensive analysis of phenolic components in *D. indica*, the total phenolic compounds extracted from *D. indica* were enriched and fractionated over a macroporous resin column; subsequently, phenolic profiling of *D. indica* was accomplished using LC-UV with two wavelengths, and individual phenolic compounds of interest were then identified by HPLC-ESI-MS/MS and ESI-IT-MS (ion trap MS). As a result, a total of 27 phenolic compounds encompassing four groups of natural phenolic components (ellagitannins, ellagic acid and ellagic acid glycosides, hydroxybenzoic acid and hydroxycinnamic acid derivatives, flavonols) were successfully identified in *D. indica*, of which except for brevifolin carboxylate (peak 6), caffeic acid (peak 7), brevifolin (peak 13), methyl brevifolin carboxylate (peak 15), ellagic acid (peak 21), and kaempferol *O-*robinobioside (peak 25) 21 phenolic compounds were identified for the first time. Furthermore, the individual phenolic compounds were then quantified based upon their LC peak areas against those of the corresponding standards, which varied from 6.69 mg per 100 g DW for ellagic acid to 71.36 mg per 100 g DW for brevifolin carboxylate. Collectively, the total contents of the four groups (ellagitannins, ellagic acid and ellagic acid glycosides, hydroxybenzoic acid and hydroxycinnamic acid derivatives, and flavonols) were 252.98, 30.52, 199.73 and 90.39 mg per 100 g DW, respectively. These quantitative data should be very helpful for future phytochemical or pharmaceutical studies on this valuable plant. In the present work, phenolic profiling of *D. indica* was achieved for the first time, which can be further developed for the characterization and evaluation of the phenolic components since they are the major active components and may be the key to the quality control for *D. indica* and its derived products. Furthermore, it is also expected that with some slight modifications, the phenolic profiling method developed in this work will be used to explore more important applications in the related biochemical or pharmaceutical research, and applied to other medicinal plants.
